# Shotgun proteomic analysis of human head and neck squamous cell carcinoma cell line SQ20B with diminished AHSG expression

**DOI:** 10.1186/1471-2105-15-S10-P35

**Published:** 2014-09-29

**Authors:** Georgina Iyamu, Pamela Thompson, Victor Paramov, Siddharth Pratap, Amos Sakwe, Josiah Ochieng, Dana Marshall

**Affiliations:** 1 School of Medicine, Meharry Medical College, Nashville, TN 37208, USA; 2 Department of Biochemistry and Cancer Biology, Meharry Medical College, Nashville, TN 37208, USA; 3 Department of Microbiology and Immunology, Meharry Medical College, Nashville, TN 37208, USA; 4 Department of Pathology, Anatomy and Cell Biology, Meharry Medical College, Nashville, TN 37208, USA

## Background

The Alpha-Heremans-Schmid Glycoprotein (AHSG) has tumor promoting properties in animal models of breast cancer and lung cancer [1,2]. These cancer cells do not synthesize AHSG, instead utilizing the liver-generated glycoprotein that is abundant in serum. We have reported that head and neck squamous cell carcinoma (HNSCC) cell lines synthesize AHSG (in press) and have also detected abundant AHSG in primary HNSCC tumors (unpublished). Growth in serum-free medium and *in vitro* tumorigenic properties, including proliferation, adhesion and migration, are diminished in the HNSCC SQ20B cell line modified with AHSG-specific shRNA to express only twenty percent of the wild-type SQ20B cell line (SQ20B-AH20) compared to SQ20B modified with empty vector alone and expressing the wild-type amount of AHSG (SQ20B-EV) [3]. Here we have used shotgun proteomic analysis to identify additional proteins whose expression may also affect these *in vitro* properties of tumorigenesis associated with AHSG.

## Materials and methods

The two cell lines were cultured in serum-free medium to avoid the contribution of exogenous AHSG in serum. Cell lysates were obtained, and the proteins were separated in polyacrylamide gels in duplicate. Each full protein lane was cut into 10 pieces, in-gel digested with trypsin and analyzed with tandem liquid chromatography mass spectrometry (LC-MS). MS data were analyzed against a recent human protein database. Proteins differentially expressed were quantified using a spectral counting approach.

## Results

**Figure 1:**
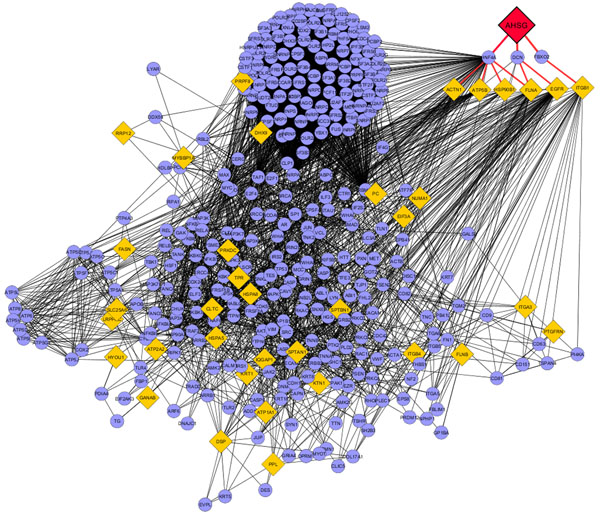
Interaction network of AHSG protein. Diamond nodes are proteins differentially expressed from samples; circular nodes are 1 degree of biological interactions.

A total of 1386 distinct protein groups were identified in the two samples. Forty-eight proteins were differentially expressed in these samples (z score > 3, which corresponds to p ≤ 0.001, and Benjamini-Hochberg FDR < 0.05). The FDR corrected seed list was analyzed using Cytoscape. Clustering within the network was based on known associations of other proteins such as HNF4α, DCN, and FBXO2 with AHSG (Figure 1).

## Conclusions

Proteins associated with the cytoskeleton, adhesion and apoptosis were over-represented in the group of differentially expressed proteins. Here we have shown that the human HNSCC cell line SQ20B exhibits prominent changes in the proteome when AHSG expression is diminished. These proteins are critical for the tumorigenic cell properties of adhesion and migration. These data will help to identify mechanisms of tumorigenesis associated with AHSG. Future work is needed to further study the role of these proteins to possibly identify new therapeutic targets for HNSCC.
